# Detection of harmful foodborne pathogens in food samples at the points of sale by MALDT-TOF MS in Egypt

**DOI:** 10.1186/s13104-021-05533-8

**Published:** 2021-03-23

**Authors:** Dalia F. Khater, Radwa A. Lela, Mohamed El-Diasty, Shawky A. Moustafa, Gamal Wareth

**Affiliations:** 1 Department of Food Hygiene, Animal Health Research Institute, Tanta Laboratory, Tanta, Egypt; 2 Animal Health Research Institute, Mansoura Provincial Laboratory, Mansoura, Egypt; 3 Faculty of Veterinary Medicine, Benha University, Moshtohor, Toukh, 13736 Egypt; 4 Friedrich-Loeffler-Institut, Institute of Bacterial Infections and Zoonoses, Jena, Germany

**Keywords:** Food Monitoring, Bacterial Hazards, Foodborne, Detection, MALDT-TOF MS, Egypt

## Abstract

**Objectives:**

Microbes can contaminate foodstuffs resulting in foodborne illnesses. Investigating microbial hazards in foods at the point of sale with rapid tools is required to avoid foodborne illness outbreaks. The current study aimed to identify the microbial hazards in food samples collected from retail shops at sale points using MALDI-TOF MS.

**Results:**

Food samples were collected from stores and supermarkets in four Delta cities (Tanta, Kutour, Kafr-Elzayat and Benha). Analysis of 178 samples of fish, meat and dairy products revealed 20 different bacterial species. 44.76% of isolates were identified as *E. coli*, 17.44% were identified as *Enterobacter* spp., and *E. cloacae* was predominant. 12.2% were identified as *Citrobacter* spp., and *C. braakii* was predominant, and 8.7% were identified as *Klebsiella* spp., and *K. pneumoniae* was predominant. Moreover, eight *Proteus mirabilis*, six *Morganella morganii*, five *Staphylococcus hominis*, three *Serratia marcescens*, two *Pseudomonas aeruginosa*, one *Salmonella typhimurium* and one *Enterococcus faecalis* were detected. Foodstuffs not only be contaminated during production and processing but also during storage and transport. Identification of harmful human pathogens in foodstuffs is alarming and consider threatening to public health. Identification of microbiological hazards in foods using MALDI-TOF MS provides an efficient tool for identifying foodborne pathogens.

## Introduction

Protection of humans from foodborne diseases is challenging. Several countries have well-developed infrastructures for monitoring food quality. However, still safe and healthy food regarding bacterial contamination a significant challenge [1]. Many microbes can contaminate foodstuffs resulting in severe health and economic implications [2]. Some are coming from the source, and some contaminate during manufacturing. Several foodborne outbreaks attributed to microbiological hazards have occurred worldwide and are associated with mortalities and economic burdens [3–5]. Thus, rapid detection of pathogenic bacteria in foodstuffs ready for human consumption is of great importance to prevent foodborne disease outbreaks and ensure food safety [6]. In Egypt, the hazard of bacterial occurrence in the food chain exists [7–9]. Methicillin-resistant *Staphylococcus aureus* (MRSA) was isolated from humans, animals and milk samples [10] and implicated in several food poisoning outbreaks [9, 11]. Several foodborne illness outbreaks associated with consuming raw or insufficiently processed foods were reported [7, 8, 11]. The highly pathogenic *E. coli* O157 and other *Enterobacteriaceae* members were recovered with a high prevalence from dairy and meat products [12] and were responsible for severe illness among patients in different governorates [13]. Multidrug resistance (MDR) *Pseudomonas aeruginosa* strains harbored resistance genes were isolated from fish farms [14]. Typhoid fever is an endemic disease in Egypt, and the infection is mainly associated with contamination of foods with *Salmonella enterica* [15]. The burden of microbiological hazards in food appeared clearly in summer 2018 after two British couple's death because of *E. coli* and *Shigella*'s, which are most prevalent in animals and associated with disease occurrence [16].

Several diagnostic tools are used for the detection of foodborne pathogens. Molecular detection of *Aeromonas hydrophila* in fish was carried out by Restriction Fragment Length Polymorphism (RFLP) [17]. PCR was applied in MRSA diagnosis [9] and detection of resistance genes [18]. Loop-mediated isothermal amplification (LAMP) was developed to detect foodborne pathogens by amplifying certain genes [19]. The current pathogen detection methods in foodstuff, either by culturing and biochemical tests or using different DNA-based assays are time consumers. They require biological culture, DNA extraction and amplification, or sequencing. However, Matrix-Assisted Laser Desorption/Ionization Time-of-Flight Mass Spectrometry (MALDI-TOF–MS) can identify the pathogen from a single colony into species level in a short time. The current study aimed to investigate the potential microbiological hazards in various food samples ready to sale for human consumption in the Delta region of Egypt and application of mass spectrometry as a useful tool for investigating foodborne pathogens.

## Main text

### Materials and methods

178 milk, meat, and fish products were collected from food stores and supermarkets in four Delta region cities during summer 2017. Ninety-two samples were collected from Tanta, the capital city of Gharbia governorate. Forty-two and thirty-six samples were collected from Kutour and Kafr-Elzayat cities of the same governorate, respectively. Eight samples were collected from Benha, the capital city of Qalyobia governorate. The samples included 108 milk products (fresh milk, cheese, yogurt, and ice cream), 47 minced meat and chicken, and 23 specimens of fish products (fillet meat and catfish) (Table 1). All food specimens were collected at points of sale. Samples were collected and transferred in sterile plastic bags within ice boxes to the Department of Food Hygiene at Animal Health Research Institute within one hour. Samples were enriched in nutrient broth. A sterile cotton swab was dipped into the broth and streaked on Columbia Agar, Columbia Nalidixic Agar (CNA), MacConkey and Mannitol Salt Agar (Oxoid Limited, Thermo Fisher Scientific, Germany). The plates were incubated at 37 °C for 24–48 h. Colonies with different morphological characteristics were harvested and subcultured on blood agar for further purification. Pure colonies of bacteria were collected by Amies agar gel with charcoal transport swabs (Thermo-Fisher Scientific, Germany) and sent directly for species identification using the MALDI-TOF MS assay.

**Table 1:** Type and number of food specimens collected from retail shops at the point of sale in four cities of Egypt's Delta region

Type of samples	No. of samples
Dairy products (n = 108)	
Fresh milk	59
Cottage cheese	25
Fresh cheese	15
Yogurt	7
Ice cream	2
Meat products (n = 47)	
Minced meat	42
Chicken meat	5
Fish products (n = 23)	
Fillet fish meat	15
Catfish	8
Total	178

Bacterial swabs were culture on blood agar media with 7,5% blood and incubated at 37° with 5% CO_2_ for 24–48 h. In a 1.5 ml Eppendorf tube, a single fresh colony from each plate was suspended in 300 µl of HPLC grade water and completely homogenized using a vortex. The bacteria were inactivated by 900 µL of absolute ethanol to each tube and then vortex again. Protein extraction from each sample was done as described before [20]. Inactivated bacterial pellets were collected by centrifugation for 2 min at 11,000*g.* The pellets were air-dried to remove ethanol traces and then were reconstituted in 50 µL of 70% formic acid and 50 µL of acetonitrile. The samples were sonicated (100% amplitude and 1.0 duty cycle) for 1 min on ice and were centrifuged at 11,290*g* for 5 min at room temperature, and the clear supernatant was collected. One µL of each supernatant was spotted onto the MALDI target (MSP 96 target polished steel (MicroScout Target) plate; Bruker Daltonik, Bremen, Germany), air-dried and overlaid with 1.0 µL of saturated α-cyano-4-hydroxycinnamic acid matrix solution (in 50% acetonitrile and 0. 25% trifluoroacetic acid). The MALDI measurements were carried out using a Microflex LT (Bruker Daltonics, Bremen, Germany) instrument and MBT Compass Explorer 4.1 software. The MALDI Biotyper manufacturer`s recommendation on the log score value of 0–3 for species identification was followed. Only bacterial species with score values equal to or more than 2.300 were considered reliably identified bacteria by MALDI-TOF MS. Isolates identified with score values less than 2.300 were excluded from the analysis.

### Results and discussion

MADLT-TOF MS successfully identified 172 bacterial isolates belonging to 20 different species. The identified bacteria included *Enterobacteriaceae* e.g. *E. coli, Salmonella typhimurium, Klebsiella* spp., *Proteus mirabilis, Enterobacter* spp., *Serratia marcescens, Citrobacter* spp., and other disease-causing bacteria (Table 2). *E. coli* was the most prevalent and represented 44.76% (n = 77) of isolates. *E. coli* was isolated from minced meat, chicken, fillet fish, fresh milk, yogurt, cottage and fresh cheese. *C. braakii, E. cloacae* and *K. pneumoniae* were representing 8.1%, 7% and 6.4%, respectively. Examination of bacterial hazards in milk samples collected from bovine revealed high incidence of *E. cloacae, K. pneumoniae, K. oxytoca, E. coli,* and *C. freundii* [21]. Examination of sixty cheese samples from retailing markets in Cairo, Giza, and Monufia revealed contamination with *E. coli* and *P. aeruginosa* in 26.6% and 1.66% of samples, respectively [22]. One strain of *S. Typhimurium* was isolated from fillet fish meat. *S. Typhimurium* is a common pathogen present in broilers flocks in Egypt [23], and contaminate chicken meat either in farm or during processing [24]. However, isolation of *S. Typhimurium* from fillet fish meat is uncommon in Egypt. 25% (43/172) of identified species were belonging to ESKAPE pathogens. ESKAPE is a group of bacterial pathogens commonly associated with MDR and encompasses *E. faecium, S. aureus, K. pneumoniae, A. baumannii, P. aeruginosa* and *Enterobacter* spp [25]*.* Thirty *Enterobacter* spp*.* (17.4%) were isolated from meat, fish and dairy products, and *E. cloacae* was predominant in 12 samples. Fifteen *Klebsiella* spp. were identified, and eleven (6.4%) were confirmed as *K. pneumoniae.* Of them, five were isolated from catfish meat, five from fresh milk samples and one from ice cream. *K. pneumoniae* is a major human pathogen that is highly common in Egyptian hospitals [26] and the environment [27]. Carbapenemase-producing *K. pneumoniae* were recovered at high frequency among broilers, drinking water and workers at poultry farms [28]. Examination of twenty brackish-water fish farms located in Damietta and Kafr-elshekh governorates revealed *K. pneumoniae, E. cloacae* and *E. coli* in 6.6%, 4.7% and 4.0% of samples, respectively [29]. Two *P. aeruginosa* were identified in milk samples obtained from Kutour city. *P. aeruginosa* was detected with a high level in camel meat [30], cheese obtained from retail markets [22], and broiler chickens and cattle [31]. *P. aeruginosa* isolates that harbored AMR genes were isolated from 31.57% of 285 examined fish samples collected from private fish farms [14]. Examination of clinical specimens collected from 133 patients at various hospitals in Cairo revealed presence of *K. pneumoniae, P. aeruginosa, E. coli* and *P. mirabilis* in 40.9%, 17.3%, 15.4% and 2.4% of samples, respectively [13]. ESKAPE pathogens are the most serious group of bacteria that can 'escape' the biocidal action of antimicrobial agents and are commonly associated with MDR patterns [25]. The presence of such notorious pathogens in fish and milk products constitutes a threat for humans.

**Table 2:** Bacterial species, geographical distribution and number of strains identified by MALDI-TOF MS in food specimens collected from retail shops at point of sale in Delta region

No	Genus and its prevalence (%)	Bacterial species	Type of food specimens	City	No. of isolates
1	*Escherichia coli* (44.76%)	*E. coli*	Minced and chicken meat, fillet fish meat, milk, yogurt, cottage, and fresh cheese	Tanta, Kutour, Kafr-Elzayat, Benha	77
2	*Enterobacter* spp. (17.44%)	*E. cloacae*	Minced meat, fillet fish meat, cottage cheese, milk	Tanta, Kutour, Kafr-Elzayat,	12
		*E. asburiae*	Fillet fish meat, minced meat, ice cream, cottage cheese	Tanta, Kutour	8
		*E. kobei*	Catfish meat, ice cream, cottage cheese	Tanta, Kafr-Elzayat	8
		*E. ludwigi*	Fillet fish	Tanta	2
3	*Citrobacter* spp. (12.2%)	*C. Freundii*	Fillet fish meat, minced meat, fresh cheese	Tanta	5
		*C. youngae*	Minced meat, yogurt	Tanta, Kafr-Elzayat	2
		*C. braakii*	Minced meat, cottage cheese	Tanta, Kafr-Elzayat	14
4	*Klebsiella* spp. (8.7%)	*K. pneumoniae*	Yogurt, catfish, milk	Kafr-Elzayat, Tanta, Kutour	11
		*k. variicola*	Yogurt, catfish	Kafr-Elzayat, Tanta	3
		*k. oxytoca*	Milk	Tanta	1
5	*Proteus* spp. (4.6%)	*P. mirabilis*	Milk	Kutour	8
6	*Morganella* spp. (3.5%)	*M. morganii*	Minced meat, milk, fresh cheese	Benha, Kutour, Tanta	6
7	*Serratia* spp. (1.7%)	*S. marcescens*	Minced meat	Benha, Tanta	3
8	*Staphylococcus* spp. (2.9%)	*S. hominis*	Minced meat, cottage cheese, yogurt	Tanta, Kafr-Elzayat	5
9	*Pseudomonas* spp. (1.1%)	*P. aeruginosa*	Milk	Kutour	2
10	*Salmonella* spp. (0.6%)	*S. Typhimurium*	Fillet fish meat	Tanta	1
11	*Enterococcus* spp. (0.6%)	*E. faecalis*	Fresh cheese	Tanat	1
12	*Raoultella* spp. (0.6%)	*R. planticola*	Minced meat	Kafr-Elzayat	1
13	*Candida* spp. (1.1%)	*Candida krusei*	Cottage cheese	Kafr-Elzayat	2
Total		20			172

Eight *Proteus mirabilis* strains were isolated from fresh milk in Kutour city. Six *Morganella morganii* strains were isolated from minced meat, fresh milk and fresh cheese in Benha, Kutour, and Tanta city. *P. mirabilis* and *P. aeruginosa* were among the most common infecting organisms causing meningitis in Egypt [32]. Three *Serratia marcescens* were isolated from minced meat samples obtained from Benha and Tanta. Several outbreaks of *S. marcescens* infection were reported in neonates in Egypt [33–35]. *M. morganii, S. marcescens*, and *P. aeruginosa* have been isolated from hospitalized patients in 2014 [36]. Microbes isolated in the current study were collected from the end products prepared for human consumption at points of sale. It is not clear if the contamination source is the animals producing food or humans, or from processing machines and the environment during manufacturing. This study highlights the potential hazard associated with foods in the Delta region of the Nile, which may transmit from food to humans through the food chain.

Several tools were used to monitor foodborne pathogens. LAMP was applied for detecting pathogens in livestock and foodstuffs [37–39], but it is less sensitive and less versatile than PCR, and a proper design of primer is a major challenge [38, 40]. Classical bacteriology is time-consuming, laborious and requires expertise. Although PCR protocols are used broadly, it is limited and identifies only the expected pathogen based on the chosen primers. MALDI-TOF is now widely used for pathogen identification in routine diagnosis [41]. It can identify microorganisms from pure colonies in minutes versus conventional methods based on culture and biochemical assays [42]. MALDI-TOF reliability and accuracy were demonstrated in numerous studies [41, 43, 44], and its use in the control of microbiological hazards in food materials will reduce the cost of consumables and time spent on other conventional diagnostics tools, giving a chance to the rapid choice of an appropriate antimicrobial therapy during food poisoning outbreaks. MALDI-TOF–MS identification is speedy, and only 2 h are required to examine a full target plate, which is considered an essential factor for food quality and safety, principally during outbreaks of food poisoning that need fast detection. Moreover, the technology requires low reagent costs represent a very substantial benefit in the food safety supply chain [45]. Application of MALDI-TOF was used successfully for microbial identification at the subspecies level in clinical diagnostic laboratories [46] and becomes the first-line diagnostic tool for microorganism identification in the last few years [47]. MALDI-TOF log score values > 2.000 were accepted in some laboratories for bacterial species identification [48]. However, the score values between 2.300 and 3.000 are considered highly probable species identification [20]. The species misidentification may be compressed the clinical outcome. In clinical laboratories, MALDI-TOF is replacing traditional diagnostic methods because it is a relatively simple technique and can overcome several challenges of identifying bacteria and fungi [49], particularly the databases containing spectra for all known microorganisms are existing.

In conclusion, Foodborne disease outbreaks can produce life-threatening illnesses in humans. To combat the burden of such outbreaks, rapid and accurate pathogen identification is necessary to choose the appropriate antimicrobial therapy rapidly. Zoonotic pathogens associated with food poisoning were determined in food products ready for human consumption at sale points. Presence of ESKAPE pathogens in foodstuffs is alarming and considers threatening public health. Pathogen identification using MALDI-TOF will provide an effective tool to identify the causes of foodborne illness and allow the physicians to apply rapid and effective antimicrobials in an optimized time to the patient.

## Limitations

Limitations are the small number of collected samples and the exclusion of all bacterial agents identified with MALDI score values less than 2.300. Antibiotic susceptibility testing for identified ESKAPE pathogens was required. A further study should be carried out to compare results of MALDT-TOF with other conventional diagnostic techniques (isolation and identification) or molecular methods using PCR.

## Data Availability

Not applicable.

## Abbreviations

MALDI TOF-MS: Matrix-assisted laser desorption ionization-time of flight mass spectrometry
*E.coli*: *Escherichia coli*
*E.*: *Enterobacter*
*C.*: *Citrobacter*
*K.*: *Klebsiella*
*P.*: *Proteus*
*S.*: *Staphylococcus*
*R.*: *Raoultella*
